# Compressing human brain activity for studying brain function

**DOI:** 10.1371/journal.pbio.3002966

**Published:** 2024-12-16

**Authors:** Bratislav Misic

**Affiliations:** Montréal Neurological Institute, McGill University, Montréal, Canada

## Abstract

What is the optimal scale or level at which to describe the brain? This Primer explores the findings of a new study published in PLOS Biology, which shows that simplifying complex brain recordings makes them more useful for studying brain function.

## Teaser

Due to its complexity and size, the optimal scale or level at which to describe the brain remains an open question in neuroscience. A new study published in *PLOS Biology* shows that simplifying complex brain recordings makes them more useful for studying brain function.

The brain is extraordinarily complex, encompassing billions of neurons. Connections among neurons promote the propagation of electrical signals, generating highly organized activity that encodes perception, cognition, and action. Propelled by exciting technological advances over the past 100 years, from the first electroencephalogram to modern high-field magnetic resonance imaging (MRI), neuroscientists have traditionally sought to record neural activity with ever-increasing detail [1]. In a new *PLOS Biology* study, Lee and colleagues flip the script, showing that broad, big picture motifs of brain activity can provide remarkably detailed information about individual people and their behavior [2].

The optimal scale or level at which to describe the brain remains an open question in neuroscience. Should we focus on single neurons, on larger areas, or on global networks? While there is a natural tendency to describe the brain from the perspective of its smallest constituent units that can be reliably measured, Lee and colleagues went in an entirely different direction, zooming out and focusing on the broader patterns in the data. Using functional MRI recordings, they identified a small, compact set of dominant patterns in moment-to-moment fluctuations of neural activity. These whole-brain motifs appear intermittently, recur perpetually over time, and can be observed in every single person.

The approach is conceptually similar to compression for multimedia. For example, although original music recordings or motion pictures are typically recorded in a high-fidelity format, the data are frequently simplified by removing sources of information (e.g., ambient noise) that do not contribute to the signal of interest (e.g., the melody). The present study takes an analogous approach, abstracting away huge of amounts of information in brain imaging recordings and bringing into focus only the most relevant elements.

Most excitingly, the methodological approach allowed the authors to go from hundreds of thousands of datapoints (“voxels” in MRI) to describe each person, to just 3, making it easier for researchers to observe important patterns across people. In this simpler, lower-dimensional space, the authors show that all participants in their study could be separated from one another. The new space simultaneously captures both patterns that generalize over time within individuals (traits) and patterns that vary over time (states). Most importantly, big data from individual scans can be summarized with a much smaller number of datapoints and used to make predictions about individual differences in behavior.

What do these findings mean for the field going forward? This simplifying framework ties together several prominent themes in neuroscience. One such theme is how to track moment-to-moment fluctuations in neural activity and relate them to ongoing thought [3]. Another is to understand how neural activity can be used to precisely describe individual-specific features and distinguish individuals from one another, akin to a fingerprint [4]. Lee and colleagues show that, by condensing relevant information and filtering out irrelevant information, fMRI recordings can help to address both questions.

A practical—but no less important—implication of this work is related to the question of optimal sample size. Multiple studies have recently shown that reliable prediction of individual differences in cognition and behavior requires thousands of fMRI scans [5], primarily because the data are so high-dimensional that one requires enough observations (individuals) to balance the number of features [6]. By significantly reducing the dimensionality of fMRI recordings from thousands to a handful, the work by Lee and colleagues effectively tips the scales and offers a more economical path forward that allows one to maintain a good observations-to-features ratio with fewer subjects. The dual state-trait representation of neural activity may therefore hold considerable promise as a framework in which to develop and test potential biomarkers.

Finally, the work of Lee and colleagues is part of a growing understanding in many natural sciences that complex systems can often be described by a compact set of underlying dimensions. In this sense, the findings provide an opportunity to render multiple data sets, including those derived using alternative recording technologies, into a common frame of reference, allowing greater cross-talk and collaboration among groups. Looking forward, it is easy to envision how a parsimonious low-dimensional description of empirical data could then be used as a platform to build and test theoretical computational models [7].

In summary, Lee and colleagues have shown that it is possible to “compress” complex brain recordings in a way to brings out their salient and most useful features. Their work offers a way to balance technological precision with methodological reductionism, raises exciting new theoretical and empirical possibilities for studying principles of brain organization.
